# Assessing the Prevalence and Pattern of Dyslipidemia Among Maintenance Hemodialysis Patients in Selected Areas of Bangladesh: A Multicenter Cross‐Sectional Study

**DOI:** 10.1002/puh2.70374

**Published:** 2026-09-10

**Authors:** Marium Sultana, Tanjina Rahman, Md. Ariful Islam, Md. Abdul Halim

**Affiliations:** ^1^ Department of Nutrition and Food Engineering Daffodil International University, DSC Dhaka Bangladesh; ^2^ Department of Food Technology and Nutrition Science Noakhali Science and Technology University Sonapur Bangladesh; ^3^ Institute of Nutrition and Food Science University of Dhaka Dhaka Bangladesh; ^4^ Department of Food and Nutrition Science Dr. Momtaz Begum University of Science and Technology Kishoreganj Bangladesh

**Keywords:** cardiovascular disease, chronic kidney disease (CKD), dyslipidemia, hemodialysis, triglyceride‐to‐HDL cholesterol ratio (TG/HDL‐C ratio)

## Abstract

**Background:**

Chronic kidney disease (CKD) is a prevalent health problem frequently associated with diabetes, hypertension, and anemia. Cardiovascular disease (CVD), particularly atherosclerotic coronary artery disease, is highly prevalent in end‐stage renal disease (ESRD), accounting for 40%–50% of deaths. Dyslipidemia, characterized by low high‐density lipoprotein cholesterol (HDL‐C) and elevated triglycerides (TG), is a common comorbidity in CKD and contributes to increased CVD risk.

**Methods:**

In this cross‐sectional study, data from 152 CKD patients undergoing maintenance hemodialysis were collected using pretested structured questionnaires, clinical assessments, and biochemical testing. The median age of participants was 48 years, with middle‐aged adults comprising 45.4% of the study population.

**Results:**

Dyslipidemia was assessed using the triglyceride‐to‐HDL cholesterol (TG/HDL‐C) ratio and the Adult Treatment Panel III (ATP III) criteria. The overall prevalence of dyslipidemia was 78.9% according to the TG/HDL‐C ratio. Dyslipidemic patients had significantly lower HDL‐C (35.9 mg/dL) and higher TG (205.5 mg/dL) compared with non‐dyslipidemic individuals. According to ATP III criteria, 79.1% of patients had mixed dyslipidemia, and 12.8% had atherogenic dyslipidemia. Patients with dyslipidemia also exhibited higher serum glucose and post‐dialysis blood urea nitrogen (BUN) levels and were more likely to be malnourished.

**Conclusion:**

Dyslipidemia is highly prevalent among CKD patients in Bangladesh, highlighting the need for early detection and targeted lipid‐lowering interventions to reduce cardiovascular risk in this population.

## Graphical abstract

This graphical abstract summarizes a multicenter cross‐sectional study of 152 maintenance hemodialysis patients in Bangladesh, showing a high prevalence of dyslipidemia (78.9%) characterized by low HDL‐C and elevated triglycerides. Most patients exhibited mixed dyslipidemia (79.1%) and atherogenic patterns (12.8%), along with higher serum glucose, increased post‐dialysis BUN, and greater risk of malnutrition and cardiovascular disease. The findings highlight the urgent need for early detection and targeted lipid‐lowering interventions to reduce cardiovascular risk in CKD patients.

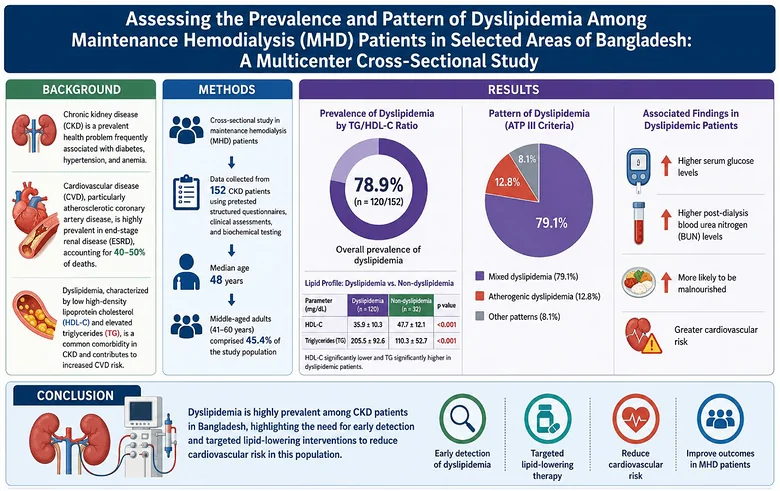

## 1 Introduction

Chronic kidney disease (CKD) is a progressive and irreversible condition characterized by a sustained decline in kidney function, representing one of the most pressing global public health challenges. Recent global estimates indicate that CKD affects approximately 10%–15% of the adult population, with its burden rising steadily across both high‐income and low‐ and middle‐income countries [1, 2]. The increase is largely driven by the growing prevalence of diabetes mellitus, hypertension, obesity, and aging populations—factors that disproportionately affect resource‐limited settings. Notably, CKD often remains clinically silent in its early stages, resulting in late diagnosis and increased progression to advanced disease stages [3].

Cardiovascular disease (CVD) remains the leading cause of mortality among individuals with CKD and end‐stage renal disease (ESRD). Contemporary evidence shows that CVD accounts for nearly 40%–50% of deaths among patients undergoing dialysis [4, 5]. The cardiovascular burden in CKD stems not only from traditional risk factors, such as hypertension, diabetes, and dyslipidemia, but also from non‐traditional CKD‐specific factors, including chronic inflammation, oxidative stress, endothelial dysfunction, vascular calcification, and the accumulation of uremic toxins [6]. These interrelated mechanisms accelerate atherosclerosis and myocardial injury, resulting in substantially increased cardiovascular morbidity and mortality.

Dyslipidemia is a central and highly prevalent metabolic abnormality in CKD and is strongly implicated in the heightened cardiovascular risk observed in this population. CKD‐associated dyslipidemia is characterized by elevated triglycerides (TG), reduced high‐density lipoprotein cholesterol (HDL‐C), impaired clearance of low‐density lipoprotein cholesterol (LDL‐C), and an increased presence of small dense LDL particles lipoprotein alterations that are markedly more atherogenic than those observed in the general population [7, 8]. The pathophysiology involves reduced lipoprotein lipase activity, impaired HDL maturation, increased hepatic very low‐density lipoprotein (VLDL) synthesis, and chronic inflammation, all contributing to disrupted lipid homeostasis [9].

Among patients with ESRD receiving maintenance hemodialysis (MHD), dyslipidemia is particularly common and clinically significant. Studies report that more than two‐thirds of MHD patients exhibit lipid abnormalities, most commonly elevated TG and low HDL‐C levels [10]. The dialysis process itself, along with chronic inflammation, oxidative stress, and exposure to dialysis membranes, further aggravates lipid disturbances [11]. Despite the high prevalence and its clear association with accelerated atherosclerosis, dyslipidemia in dialysis patients remains underdiagnosed and inadequately managed, particularly in low‐resource settings where routine lipid monitoring is limited.

Regional evidence further demonstrates the substantial burden of lipid abnormalities among patients receiving hemodialysis, although prevalence estimates vary according to the lipid parameters and diagnostic thresholds applied. In a Bangladeshi study of 31 hemodialysis patients, elevated TG, reduced HDL‐C, elevated LDL‐C, and elevated total cholesterol (TC) were observed in 83.9%, 80.7%, 77.4%, and 71.0% of patients, respectively [12]. A cross‐sectional study involving 153 hemodialysis patients in Syria reported reduced HDL‐C in 72.5% of participants and elevated TG in 37.3% [13]. More recently, a large cohort study involving 50,673 incident hemodialysis patients demonstrated that the triglyceride‐to‐HDL cholesterol ratio (TG/HDL‐C ratio) was associated with all‐cause and cardiovascular mortality, suggesting its potential role as a marker of metabolic and cardiovascular risk in patients receiving MHD [14].

Nutritional status plays a crucial role in shaping lipid profiles in CKD, and malnutrition, often termed protein‐energy wasting, is a common complication in advanced CKD and MHD patients. Factors, such as dietary restrictions, poor appetite, inflammation, metabolic acidosis, and nutrient losses, during dialysis contribute to protein‐energy wasting, which in turn affects lipid metabolism by lowering HDL‐C, elevating TG, and altering lipoprotein composition [15]. The coexistence of malnutrition and dyslipidemia further increases cardiovascular vulnerability, creating a complex clinical scenario requiring multidimensional assessment. Given the growing CKD burden and the amplified cardiovascular risk associated with dyslipidemia and poor nutritional status, understanding the patterns and determinants of lipid abnormalities in MHD patients is essential. Such insights are crucial for guiding early intervention, individualized nutritional care, and evidence‐based lipid management strategies, particularly in regions with limited healthcare resources.

Although dyslipidemia in CKD has been extensively studied in Western populations, data from South Asia, including Bangladesh, are limited. Bangladesh has experienced a rapid increase in non‐communicable diseases, such as diabetes and hypertension, which are major risk factors for CKD. The prevalence and patterns of dyslipidemia in Bangladeshi MHD patients, and their relationship with dietary intake and nutritional status, remain poorly characterized. Addressing this knowledge gap is crucial for developing context‐specific guidelines for lipid management and cardiovascular risk reduction in this high‐risk population.

This study aimed to investigate the prevalence and patterns of dyslipidemia among MHD patients in Bangladesh. Specifically, we assessed lipid abnormalities using both the TG/HDL‐C ratio and the Adult Treatment Panel III (ATP III) guidelines. Additionally, we examined associations between dyslipidemia and dietary intake of macro‐ and micronutrients to explore potential nutritional contributors to lipid abnormalities. By providing insights into the burden and determinants of dyslipidemia in this population, this study seeks to inform clinical practice and public health strategies aimed at reducing cardiovascular risk among CKD patients in Bangladesh.

## 2 Materials and Methods

### 2.1 Study Design and Setting

This study employed a cross‐sectional analytical design to assess lipid abnormalities among adult patients undergoing MHD in Bangladesh. Data were collected from four districts—Cumilla, Dhaka, Mymensingh, and Noakhali—to capture variation in demographic and clinical characteristics across different geographic locations. Recruitment was carried out in established hemodialysis centers that routinely provide dialysis services to patients with ESRD. The study was conducted over a defined period of clinical visits to ensure that all measurements and interviews were performed in a standardized and consistent manner.

### 2.2 Study Population and Sampling Strategy

A total of 152 adult MHD patients were enrolled using a convenience sampling approach, in which all eligible patients attending the participating dialysis centers during the data‐collection period were invited to participate. This method was selected due to the practical and ethical constraints of working with a medically vulnerable population undergoing fixed hemodialysis schedules. Convenience sampling is commonly applied in nephrology research when working with specialized patient groups with restricted availability. Although no formal a priori sample‐size calculation was conducted, the sample provides acceptable precision for descriptive estimates. At a 95% confidence level and assuming a conservative prevalence estimate of *p* = 0.5, the maximum margin of error is approximately ±8%, indicating reasonable accuracy for estimating population proportions.

### 2.3 Eligibility Criteria

#### 2.3.1 Inclusion Criteria

Participants were eligible if they were older than 18 years, diagnosed with ESRD, and receiving MHD at least twice per week. Only patients who had been on hemodialysis for ≥3 consecutive months were included to ensure clinical stability and minimize the influence of acute fluctuations in lipid metabolism. Participants were also required to be physically and cognitively able to provide written informed consent.

#### 2.3.2 Exclusion Criteria

Patients were excluded if they declined participation, had incomplete or missing biochemical or clinical data at the time of data collection, or were experiencing acute infection, recent hospitalization, or clinical instability, as such conditions could alter metabolic or lipid parameters and compromise the accuracy of the study.

### 2.4 Data Collection Procedures

Data collection was conducted from February 7, 2025 to May 3, 2025. Eligible patients were approached, and their demographic, clinical, anthropometric, biochemical, and dietary information was recorded. A validated, pretested questionnaire was administered to obtain demographic variables (age, sex, and residence), clinical information (duration of dialysis, comorbidities, medications, and dialysis status), and lifestyle factors. Biological data, including fasting lipid profiles (TC, TG, HDL‐C, and LDL‐C), renal function markers, electrolytes, and other biochemical parameters, were extracted from recent laboratory reports generated by standard automated analyzers at the dialysis centers. Anthropometric measurements included height, pre‐ or post‐dialysis weight, body mass index (BMI), mid‐arm circumference (MAC), mid‐arm muscle circumference (MAMC), and triceps skinfold thickness (TSF). Clinical parameters, such as blood pressure, diabetes status, malnutrition‐inflammation score (MIS), restless leg syndrome (RLS), dialysis adequacy test (ADAT), hepatitis status, and transplant history, were also recorded. Dietary intake was assessed using a 3‐day food diary, following KDOQI recommendations for CKD populations. To better capture habitual dietary intake and account for potential variations in appetite associated with the hemodialysis treatment schedule, participants were instructed to record dietary intake across both dialysis and non‐dialysis days whenever feasible. Following completion of data collection, all information was entered into SPSS version 26 starting from May 5, 2025, and subsequent data cleaning, verification, and analysis were performed gradually to ensure accuracy and completeness.

### 2.5 Classification of Dyslipidemia

Dyslipidemia was classified on the basis of the triglyceride (TG) to HDL‐C ratio, an established marker of atherogenic risk and metabolic disturbances in MHD patients. Participants were categorized as dyslipidemic if the TG/HDL‐C ratio was >3.8 and non‐dyslipidemic if the ratio was ≤3.8. This cutoff is widely used in nephrology and cardiovascular research because of its strong predictive value for insulin resistance, small dense LDL formation, and endothelial dysfunction, conditions commonly observed in patients undergoing chronic hemodialysis.

### 2.6 Measurements

Anthropometric: height, pre‐ and post‐dialysis weight, BMI, MAC, MAMC, and TSF.

Clinical: blood pressure, diabetes status, MIS, RLS, ADAT, dialysis status, hepatitis status, and transplant history.

Biochemical: creatinine (Cr), glucose (Glu), hemoglobin (Hb), albumin (Alb), sodium (Na), potassium (K), phosphorus (P), total iron‐binding capacity (TIBC), pre‐ and post‐dialysis blood urea nitrogen (BUN), and lipid profiles, including TC, TAG, HDL‐C, and LDL‐C.

Dietary: a 3‐day food diary, recorded according to KDOQI recommendations for CKD populations.

### 2.7 Statistical Analysis

Data were analyzed using SPSS version 26. Normality of continuous variables was assessed using the Kolmogorov–Smirnov test. Normally distributed data were expressed as mean ± standard deviation (SD), whereas non‐normally distributed data were presented as median (interquartile range, IQR). Group comparisons were performed using independent *t*‐tests or Mann–Whitney *U* tests, as appropriate. Correlations were assessed with Pearson or Spearman correlation coefficients. Categorical variables were analyzed using chi‐square or Fisher's exact tests. A *p* value <0.05 was considered statistically significant.

### 2.8 Ethical Considerations

Ethical clearance was obtained from the Institutional Review Board (IRB) of Daffodil International University (DIU) (Ref.: FAUSREC/DIU/2025/SMIG‐04). All participants were informed about the study and the IRB approval, and written informed consent was obtained before enrollment. All procedures were conducted in accordance with the principles of the Declaration of Helsinki, and participant confidentiality was strictly maintained.

## 3 Results

### 3.1 Study Population Characteristics

This cross‐sectional study included 152 MHD patients, of whom 55.3% (*n* = 84) were male and 44.7% (*n* = 68) were female. The primary causes of CKD in this population were hypertension (38.8%), combined hypertension and diabetes (25%), unknown etiologies (25%), glomerulonephritis (3.9%), hypertensive‐glomerulonephritis (3.9%), and diabetes alone (3.3%). These findings indicate that hypertension, alone or in combination with diabetes, remains the leading contributor to CKD in this cohort, consistent with global and regional trends in CKD etiology.

The median age of participants was 48 years (IQR 32.2–58.5), with nearly half (48.7%) in the “at‐risk” age group (middle‐aged adults), and the highest prevalence observed in middle‐aged adults (45.4%). The median estimated glomerular filtration rate (eGFR) was markedly reduced at 5.3 mL/min/1.73 m^2^ (IQR 3.9–7.1), confirming the advanced renal dysfunction of this cohort. Dialysis session characteristics showed a median duration of 4 h per session (IQR 3.5–4) and a dialysis vintage of 23 months (IQR 12.25–43.75).

Regarding dialysis modalities, polysulfone dialyzers were used in 57.9% of patients and polynephrones in 42.1%, with bicarbonate‐based dialysate used in 73% of cases. Dialysis was predominantly performed twice weekly (98%), reflecting common practice patterns in Bangladesh. Dialyzer reuse occurred in 58.9% of patients. All participants had arteriovenous fistula (AVF) as their vascular access, indicating adherence to recommended vascular access guidelines. Socioeconomic data showed that 11.8% of patients were from very poor households, 21.7% poor, 37.5% moderate, and 28.9% high‐income households (Table 1).

**TABLE 1:** Demographic and clinical characteristics of hemodialysis patients (*n* = 152).

Parameters	Total (*n* = 152)
**Age, years (median, IQR)**	48 (35.25–58.5)
**Age risk group**	
At risk	74 (48.7%)
No risk	78 (51.3%)
**Age type**	
Young adult	37 (24.3%)
Middle age adult	69 (45.4%)
Older adult	46 (30.3%)
**eGFR, mL/min/1.73 m^2^ (median, IQR)**	5.3 (3.9–7.1)
**Dialysis duration, h (median, IQR)**	4 (3.5–4)
**Dialysis vintage, months (median, IQR)**	23 (12.25–43.75)
**Dialyzer type**	
Polysulfone	88 (57.9%)
Polynephrones	64 (42.1%)
**Dialysate buffer**	
Bicarbonate	111 (73%)
Acetate	41 (27%)
**Dialysis frequency**	
Twice a week	149 (98%)
Thrice a week	3 (2%)
**Household income**	
Very poor	18 (11.8%)
Poor	33 (21.7%)
Moderate	57 (37.5%)
High	44 (28.9%)
**Dialyzer reuse**	
Yes	85 (58.9%)
No	67 (41.1%)
**Vascular access type**	AVF 152 (100%)

Abbreviations: AVF, arteriovenous fistula; eGFR, estimated glomerular filtration rate; IQR, interquartile range.

### 3.2 Prevalence and Patterns of Dyslipidemia

Dyslipidemia was highly prevalent in this MHD population. On the basis of the triglyceride (TG) to HDL‐C ratio, 78.9% of patients had dyslipidemia (TG/HDL‐C >3.8). Among these, 82.1% were male and 75% female, but the difference was not statistically significant (*χ* ^2^ = 1.154, *p* = 0.283; Table 2). When classified according to the ATP III guidelines, 13.1% of patients had atherogenic dyslipidemia (meeting all three criteria: low HDL, high LDL, and high TG), 80.4% had mixed dyslipidemia (meeting one or two criteria), and 5.9% had normal lipid profiles. No significant sex differences were observed, indicating a broadly similar distribution of dyslipidemia patterns across males and females (Table 3).

**TABLE 2:** Prevalence of dyslipidemia on the basis of TG/HDL‐C ratio.

Category	TG/HDL‐C	Total *n* (%)	Male *n* (%)	Female *n* (%)	*χ* ^2^	*p* value
Dyslipidemia	>3.8	120 (78.9)	69 (82.1)	51 (75)	1.154	0.283
No dyslipidemia	≤3.8	32 (21.1)	15 (17.9)	17 (25)	—	—

Abbreviation: TG/HDL‐C, triglyceride‐to‐high‐density lipoprotein cholesterol.

**TABLE 3:** Pattern of dyslipidemia according to Adult Treatment Panel III (ATP III) guideline.

Type	Criteria	Total *n* (%)	Male *n* (%)	Female *n* (%)	*χ* ^2^	*p* value
Atherogenic	HDL < 40 mg/dL, LDL > 100 mg/dL, TG > 150 mg/dL	20 (13.1)	14 (16.7)	6 (8.8)	2.31	0.315
Mixed dyslipidemia	HDL < 40 mg/dL + LDL > 100 mg/dL OR HDL < 40 mg/dL + TG > 150 mg/dL	123 (80.4)	66 (78.6)	57 (83.8)	—	—
Normal	HDL > 40 mg/dL, LDL < 100 mg/dL, TG < 150 mg/dL	9 (5.9)	4 (4.8)	5 (7.4)	—	—

Abbreviations: HDL, high‐density lipoprotein; LDL, low‐density lipoprotein.

Spearman correlation analysis revealed a significant positive association between the TG/HDL‐C ratio and ATP III guideline classifications (*r* = 0.414, *p* < 0.001), demonstrating that both methods reliably capture dyslipidemic status in MHD patients. Furthermore, *χ* ^2^ testing confirmed statistically significant concordance between the two methods (*p* < 0.001), suggesting that either method could be used clinically for dyslipidemia screening in this population (Table 4).

**TABLE 4:** Correlation between dyslipidemia assessment methods (Spearman).

Methods	DL: TG/HDL‐C	DL: ATP III guideline
DL: TG/HDL‐C	—	*r* = 0.414, *p* = 0.000
DL: ATP III guideline	*r* = 0.414, *p* = 0.000	—

Abbreviations: ATP III, Adult Treatment Panel III; TG/HDL‐C, triglyceride‐to‐high‐density lipoprotein cholesterol.

### 3.3 Dietary Intake Analyses

#### 3.3.1 Macronutrient Intake

Daily macronutrient intake was assessed to explore potential dietary contributors to dyslipidemia. Median energy intake was 1463.6 kcal/day (IQR 1250.6–1654.3), protein intake 57.6 g/day (IQR 46.4–64.9), carbohydrate intake 211.3 g/day (IQR 176.9–237.2), and total fat 40.6 g/day (IQR 31.1–50.7). Saturated (SFA), monounsaturated (MUFA), and polyunsaturated (PUFA) fat intakes showed no significant differences between patients with or without dyslipidemia, regardless of classification method. Total dietary fiber intake averaged 14.6 ± 6.3 g/day (Table 5). These findings suggest that dietary macronutrient intake may not directly explain the high prevalence of dyslipidemia in this cohort.

**TABLE 5:** Macronutrient intake in hemodialysis patients by dyslipidemia type.

Nutrient	All patients	TG/HDL‐C >3.8 (*n* = 120)	TG/HDL‐C ≤3.8 (*n* = 32)	*p* value	ATP III: AD (*n* = 20)	MD (*n* = 123)	No DL (*n* = 9)	*p* value
Energy (kcal)	1463.6 (1250.6–1654.3)	1496.1 (1311.4–1657.3)	1442.3 (1089.9–1604.3)	0.185	1471.8 (1329.4–1676.2)	1470.5 (1247.8–1657.9)	1437.4 (998.7–1529.1)	0.675
Protein (g)	57.6 (46.4–64.9)	57.9 (47.3–65.0)	54.8 (40.4–64.3)	0.292	57.6 (46.8–63.5)	58.0 (47.1–65.7)	52.2 (39.3–60.3)	0.866
Carbohydrate (g)	211.3 (176.9–237.2)	215.9 (179.9–238.0)	196.9 (155.8–228.6)	0.132	219.6 (188.1–236.9)	210.9 (176.6–238.4)	194.3 (140.0–227.0)	0.503
Fat (g)	40.6 (31.1–50.7)	41.2 (31.9–51.0)	38.7 (28.0–49.9)	0.327	38.7 (28.7–46.5)	41.2 (31.8–52.6)	39.1 (25.9–53.7)	0.474
SFA (g)	1.4 (0.78–2.9)	1.5 (0.7–3.4)	1.4 (0.9–2.3)	0.864	1.2 (0.5–2.8)	1.5 (0.8–3.8)	1.2 (0.8–1.7)	0.376
MUFA (g)	1.2 (0.7–2.2)	1.2 (0.7–2.3)	1.3 (0.9–2.0)	0.765	1.0 (0.6–3.1)	1.2 (0.7–2.3)	1.2 (0.7–1.4)	0.402
PUFA (g)	0.5 (0.3–1.5)	0.5 (0.3–1.4)	0.6 (0.4–2.0)	0.659	0.4 (0.2–1.7)	0.6 (0.3–1.6)	0.6 (0.3–0.7)	0.370
Fiber (g)	14.56 ± 6.26	14.78 ± 6.38	13.7 ± 5.8	0.411	14.95 ± 6.34	14.41 ± 6.35	15.77 ± 5.18	0.788

Abbreviations: AD, atherogenic dyslipidemia; ATP III, Adult Treatment Panel III; MD, mixed dyslipidemia; MUFA, monounsaturated fat; No DL, no dyslipidemia; PUFA, polyunsaturated fat; SFA, saturated fat; TG/HDL‐C, triglyceride‐to‐high‐density lipoprotein cholesterol.

#### 3.3.2 Micronutrient Intake

Analyses of both vitamins and minerals revealed no statistically significant differences between dyslipidemic and non‐dyslipidemic patients (Tables 6 and 7). Median intakes of fat‐soluble vitamins (A, D, E, and K) and water‐soluble vitamins (B1, B2, B3, B6, B9, B12, and C) were similar across groups. Mineral intakes, including phosphorus, sodium, potassium, calcium, magnesium, zinc, and iron, also showed minor differences between sexes: males consumed more phosphorus, sodium, potassium, magnesium, zinc, and iron, whereas females had slightly higher calcium and cholesterol intake. However, none of these differences reached statistical significance, suggesting that variations in micronutrient intake were not a major determinant of dyslipidemia in this population.

**TABLE 6:** Micronutrient (vitamins) intake based on dyslipidemia.

Nutrient	All patients	TG/HDL‐C >3.8 (*n* = 120)	TG/HDL‐C ≤3.8 (*n* = 32)	*p* value	ATP III: AD (*n* = 20)	MD (*n* = 123)	No DL (*n* = 9)	*p* value
**Fat‐soluble vitamins**								
Vitamin A (IU)	454.6 (314.5–853.3)	450.6 (314.5–870.5)	487.4 (305.1–697.4)	0.982	461.1 (319.0–825.7)	476.1 (315.7–877.8)	328.2 (158.4–556.8)	0.780
Vitamin D (IU)	4.4 (0.7–11.3)	4.4 (0.7–11.7)	4.7 (0.4–10.9)	0.993	4.3 (0.7–18.9)	4.4 (0.7–11.8)	4.4 (0.5–9.5)	0.874
Vitamin E (mg)	6.6 (4.0–8.3)	6.8 (4.2–8.5)	5.3 (4.0–7.4)	0.136	6.9 (4.5–8.5)	6.7 (3.4–8.3)	5.8 (4.4–8.1)	0.718
Vitamin K (mg)	2.2 (1.4–4.0)	2.2 (1.4–4.1)	2.3 (1.5–3.8)	0.874	2.5 (1.9–3.7)	2.2 (1.4–4.2)	1.6 (0.8–2.6)	0.629
**Water‐soluble vitamins**								
Vitamin B1 (mg)	0.8 ± 0.3	0.8 ± 0.3	0.7 ± 0.3	0.292	0.8 ± 0.3	0.8 ± 0.3	0.8 ± 0.2	0.747
Vitamin B2 (mg)	0.8 (0.6–1.0)	0.8 (0.6–1.0)	0.8 (0.6–1.0)	0.287	0.8 (0.6–1.2)	0.8 (0.6–1.0)	0.8 (0.8–1.0)	0.710
Vitamin B3 (mg)	17.2 (10.8–22.0)	17.6 (11.8–22.1)	15.3 (9.6–21.2)	0.387	17.6 (11.5–20.2)	17.1 (9.7–22.6)	16.1 (13.4–20.9)	0.811
Vitamin B6 (mg)	0.8 (0.6–0.9)	0.8 (0.6–0.9)	0.5 (0.8–0.9)	0.671	0.7 (0.6–0.8)	0.8 (0.6–1.0)	0.8 (0.5–0.9)	0.474
Vitamin B9 (µg)	125.2 (88.4–160.6)	125.3 (89.3–160.8)	110.1 (77.3–159.7)	0.841	121.15 (94.21–152.30)	125.3 (87.6–165.8)	110.8 (95.4–158.1)	0.958
Vitamin B12 (µg)	0.2 (0.08–0.4)	0.2 (0.09–0.4)	0.3 (0.03–0.4)	0.586	0.272 (0.086–0.485)	0.2 (0.09–0.4)	0.2 (0.02–0.4)	0.995
Vitamin C (mg)	50.1 (32.8–87.9)	50.1 (32.9–81.5)	54.4 (32.3–107.5)	0.651	49.01 (34.08–100.4)	50.2 (32.8–78.5)	85.6 (31.3–142.2)	0.977

Abbreviations: AD, atherogenic dyslipidemia; ATP III, Adult Treatment Panel III; DL, dyslipidemia; MD, mixed dyslipidemia; TG/HDL‐C, triglyceride‐to‐high‐density lipoprotein cholesterol.

**TABLE 7:** Micronutrient (minerals) intake based on dyslipidemia.

Mineral	All patients	TG/HDL‐C >3.8 (*n* = 120)	TG/HDL‐C ≤3.8 (*n* = 32)	*p* value	ATP III: AD (*n* = 20)	MD (*n* = 123)	No DL (*n* = 9)	*p* value
Phosphorus (mg)	774.0 (571.0–913.1)	787.9 (591.7–919.8)	701.4 (558.9–847.4)	0.235	827.4 (579.5–932.7)	766.3 (565.6–913.2)	711.0 (601.1–862.7)	0.492
Sodium (mg)	1825.4 (1256.2–2218.3)	1894.4 (1265.3–2249.9)	1507.5 (1231.7–2129.5)	0.284	1944.4 (1140.4–2327.4)	1813.1 (1262.6–2188.2)	1722.2 (1173.0–2214.8)	0.839
Potassium (mg)	1480.1 (1060.1–1874.3)	1511.6 (1089.1–1898.1)	1336.5 (985.7–1752.8)	0.376	1592.6 (1036.5–1795.5)	1462.0 (1020.5–1875.8)	1441.3 (1211.5–1934.8)	0.753
Calcium (mg)	311.7 (228.9–421.4)	321.1 (221.8–435.5)	302.3 (237.9–379.7)	0.515	395.2 (224.4–503.6)	311.8 (225.6–411.8)	280.9 (253.9–391.2)	0.284
Magnesium (mg)	216.1 ± 84.7	219.7 ± 86.6	202.6 ± 77.2	0.314	223.9 ± 85.6	213.7 ± 86.7	231.6 ± 54.7	0.754
Zinc (mg)	6.6 ± 2.5	6.7 ± 2.5	6.2 ± 2.4	0.292	6.7 ± 2.0	6.6 ± 2.6	6.3 ± 1.4	0.932
Iron (mg)	10.6 (7.6–14.5)	11.5 (7.7–15.3)	9.4 (7.2–12.8)	0.097	11.6 (6.8–23.9)	10.4 (7.6–14.5)	9.8 (8.3–12.4)	0.419
Cholesterol (mg)	78.3 (19.7–139.8)	67.2 (26.2–140.1)	80.5 (11.5–138.2)	0.750	68.7 (27.8–144.8)	79.5 (19.5–139.8)	62.0 (6.9–129.4)	0.965

Abbreviations: AD, atherogenic dyslipidemia; ATP III, Adult Treatment Panel III; DL, dyslipidemia; MD, mixed dyslipidemia; TG/HDL‐C, triglyceride‐to‐high‐density lipoprotein cholesterol.

### 3.4 Association Between Nutrient Intake and Dyslipidemia

Binary logistic regression was conducted to evaluate the impact of nutrient intake on dyslipidemia. Higher energy intake was associated with a 1.04‐fold increased likelihood of dyslipidemia (95% CI 0.958–1.13), and higher saturated fat intake with a 1.01‐fold increase, although these associations were not statistically significant. Similarly, other macronutrients, vitamins, and minerals did not show significant effects on dyslipidemia prevalence (Table 8). These results suggest that dyslipidemia in MHD patients may be influenced more by metabolic and dialysis‐related factors than by dietary intake alone.

**TABLE 8:** Association between nutrient intake and dyslipidemia (binary logistic regression).

Nutrient	Dyslipidemia TG/HDL‐C >3.8 (*n* = 120)	≤3.8 (*n* = 32)	Adjusted odds ratio (AOR)	95% CI	*p* value
Energy (kcal)	1496.1 (1311.4–1657.3)	1442.3 (1089.9–1604.3)	1.04	0.958–1.13	0.340
Carbohydrates (g)	215.9 (179.9–238.0)	196.9 (155.8–228.6)	0.859	0.615–1.19	0.369
Protein (g)	57.9 (47.3–65.0)	54.8 (40.4–64.3)	0.864	0.598–1.24	0.434
Fat (g)	41.2 (31.9–51.0)	38.7 (28.0–49.9)	0.700	0.341–1.43	0.332
SFA (g)	1.5 (0.76–3.4)	1.4 (0.9–2.3)	1.01	0.692–1.47	0.956
MUFA (g)	1.2 (0.7–2.3)	1.3 (0.9–2.0)	0.949	0.321–2.80	0.924
PUFA (g)	0.5 (0.3–1.4)	0.6 (0.4–2.0)	0.932	0.617–1.40	0.736
Total Fiber (g)	14.7 ± 6.3	13.7 ± 5.8	0.889	0.713–1.10	0.296
Cholesterol (mg)	67.2 (26.2–140.1)	80.5 (11.5–138.2)	0.999	0.989–1.01	0.924
Sodium (mg)	1894.4 (1265.3–2249.9)	1507.5 (1231.7–2129.5)	1.0	0.999–1.00	0.686
Potassium (mg)	1511.6 (1089.1–1898.1)	1336.5 (985.7–1752.8)	0.999	0.997–1.00	0.696
Phosphorus (mg)	787.9 (591.7–919.8)	701.4 (558.9–847.4)	0.999	0.994–1.00	0.707
Iron (mg)	11.5 (7.7–15.3)	9.4 (7.2–12.8)	1.013	0.982–1.04	0.429
Magnesium (mg)	219.7 ± 86.6	202.6 ± 77.2	1.0	0.980–1.02	0.990

Abbreviations: MUFA, monounsaturated fat; PUFA, polyunsaturated fat; SFA, saturated fat; TG/HDL‐C, triglyceride‐to‐high‐density lipoprotein cholesterol.

## 4 Discussion

This multicenter cross‐sectional study provides a comprehensive assessment of the prevalence, patterns, and potential nutritional correlates of dyslipidemia among MHD patients in selected areas of Bangladesh. The findings demonstrate a very high burden of dyslipidemia in this population, with nearly four‐fifths of patients affected, and reveal a characteristic lipid pattern dominated by elevated triglycerides and low HDL‐C. Importantly, dietary intake of macro‐ and micronutrients showed no significant association with dyslipidemia, suggesting that metabolic and dialysis‐related factors play a more dominant role in lipid abnormalities among MHD patients.

### 4.1 Demographic and Clinical Profile of the Study Population

The relatively young median age (48 years) observed in this cohort is notable and contrasts with dialysis populations in high‐income countries, where the median age often exceeds 60 years. This younger age distribution likely reflects earlier onset of CKD and more rapid progression to ESRD in Bangladesh, driven by poorly controlled hypertension and diabetes, delayed diagnosis, and limited access to preventive nephrology services. Younger dialysis populations still face disproportionately high cardiovascular risk, making early identification of modifiable risk factors, such as dyslipidemia, particularly important. All participants had advanced renal dysfunction, with a median eGFR of 5.3 mL/min/1.73 m^2^, confirming ESRD status. Dialysis characteristics, including predominantly twice‐weekly dialysis, dialyzer reuse, and mixed dialyzer membrane types, reflect real‐world practice in resource‐limited settings. They may influence lipid metabolism through effects on inflammation, oxidative stress, and uremic toxin clearance. Previous studies have shown that inadequate dialysis dose and membrane bio‐incompatibility can exacerbate inflammation and dyslipidemia in MHD patients [6].

### 4.2 High Prevalence of Dyslipidemia and Atherogenic Lipid Pattern

The high prevalence of dyslipidemia on the basis of the TG/HDL‐C ratio (78.9%) underscores the magnitude of atherogenic lipid abnormalities in this MHD cohort. The TG/HDL‐C ratio has gained increasing attention as a robust surrogate marker of atherogenic dyslipidemia, insulin resistance, and the presence of small dense LDL particles features that are highly prevalent in CKD and ESRD and that are not always captured by conventional lipid measures alone. Recent observational evidence suggests a significant association between elevated TG/HDL‐C and CKD risk and progression, with higher ratios correlating with worsening renal function and increased cardiometabolic risk [7, 16, 17]. A large cross‐sectional study in a Chinese population also demonstrated a positive and nonlinear relationship between TG/HDL‐C ratio and CKD prevalence, indicating that higher ratios are independently linked to greater CKD risk [18].

The present findings are broadly consistent with studies from neighboring South Asian countries and other developing regions. Studies conducted among MHD patients in India and Pakistan have similarly reported a characteristic pattern of elevated triglycerides and reduced HDL‐C, whereas TC and LDL‐C levels were often normal or only modestly altered [19, 20, 21]. These findings support the predominance of triglyceride‐rich, low‐HDL‐C dyslipidemia rather than isolated LDL‐C elevation among South Asian patients receiving hemodialysis.

Comparable patterns have also been observed in other developing regions. A study from Iraq reported an elevated TG/HDL‐C ratio in 64.5% of MHD patients [22], which was lower than the prevalence of 78.9% observed in the present cohort. Another study from the Kurdistan region of Iraq reported a high atherogenic index in 84% of hemodialysis patients, with hypertriglyceridemia and low HDL‐C present in approximately half of the study population [23]. Differences in prevalence across studies may be related to variations in the definition of dyslipidemia, demographic characteristics, diabetes burden, nutritional status, dialysis duration and frequency, medication use, and access to healthcare. Nevertheless, the overall consistency of the lipid pattern suggests that atherogenic dyslipidemia is a common metabolic abnormality among hemodialysis patients in resource‐limited settings.

The dominance of elevated triglycerides and reduced HDL‐C in ESRD reflects profound disturbances in lipoprotein metabolism. Hypertriglyceridemia results from decreased activity of lipoprotein lipase and hepatic lipase, leading to impaired clearance of triglyceride‐rich lipoproteins, such as VLDL and chylomicron remnants. Simultaneously, HDL‐C levels decline due to reduced synthesis of apolipoprotein A‐I and defective HDL maturation, and the functional quality of HDL particles is further compromised by chronic inflammation and oxidative stress hallmarks of uremic milieu which impair reverse cholesterol transport and antioxidant properties of HDL particles. These structural and functional alterations contribute to increased cardiovascular risk in CKD patients by promoting endothelial dysfunction, inflammation, and atherogenesis [9, 15]. Elevated TG/HDL‐C ratio therefore not only reflects quantitative lipid abnormalities but also captures qualitative dyslipidemic features that have important clinical implications for risk stratification in ESRD populations. In low‐resource settings like Bangladesh, where comprehensive lipid profiling may not be routinely available, the TG/HDL‐C ratio could serve as a pragmatic and accessible marker for early identification of high‐risk individuals and for guiding preventive strategies.

### 4.3 ATP III–Defined Dyslipidemia and Clinical Interpretation

According to ATP III criteria, mixed dyslipidemia was the predominant pattern (80.4%), whereas atherogenic dyslipidemia affected 13.1% of patients. Only a small minority (5.9%) had normal lipid profiles. Mixed dyslipidemia characterized by low HDL‐C in combination with elevated triglycerides and/or increased LDL‐C is clinically significant because it confers a greater cardiovascular risk than isolated lipid abnormalities [24]. This pattern is consistent with previous studies in patients with CKD and ESRD, where reduced HDL‐C and hypertriglyceridemia are reported as the most frequent lipid disturbances [25, 26, 27]. Mikolasevic et al. [27] highlighted that dyslipidemia in dialysis patients is predominantly marked by low HDL‐C and elevated triglycerides rather than isolated LDL‐C elevation. Similarly, Blaton [28] described CKD‐associated dyslipidemia as primarily driven by impaired triglyceride metabolism and defective HDL maturation due to reduced lipoprotein lipase activity, altered apolipoprotein metabolism, and accumulation of uremic toxins. These CKD‐specific metabolic alterations explain the high burden of mixed and atherogenic dyslipidemia observed in advanced renal disease. Furthermore, the absence of significant sex differences in dyslipidemia categories in our study aligns with findings from other dialysis cohorts, where hormonal influences on lipid metabolism appear attenuated in the presence of uremia [27, 29].

### 4.4 Concordance Between TG/HDL‐C Ratio and ATP III Criteria

The significant correlation between the TG/HDL‐C ratio and ATP III classification (*r* = 0.414, p < 0.001) indicates moderate agreement between these two methods for assessing dyslipidemia patterns in MHD patients. This finding supports the clinical utility of the TG/HDL‐C ratio as a simple and cost‐effective screening tool, particularly in settings where full lipid profiles may not be routinely available. A growing body of evidence suggests that an elevated TG/HDL‐C ratio reflects underlying atherogenic dyslipidemia and metabolic risk in CKD and other cardiometabolic populations. A recent cross‐sectional study in MHD patients reported that a high TG/HDL‐C ratio was prevalent in a majority of participants and underscored its value as an indicator of dyslipidemia beyond conventional LDL‐based measures, which may be misleading in dialysis populations [30]. Moreover, large observational evidence and systematic analyses have demonstrated that a higher TG/HDL‐C ratio is associated with increased risk of CKD progression and elevated cardiometabolic risk across diverse populations, highlighting its potential as a practical surrogate marker in clinical practice [31, 32]. In low‐resource settings like Bangladesh, where access to comprehensive lipid testing may be limited, incorporating the TG/HDL‐C ratio into routine evaluation could help identify high‐risk individuals early and guide preventive strategies.

### 4.5 Dietary Intake and Dyslipidemia: Explaining the Null Findings

A key strength of this study is the comprehensive assessment of dietary intake; however, no significant associations were identified between dyslipidemia and intake of energy, macronutrients, fatty acid subtypes, fiber, vitamins, or minerals. Logistic regression analysis further confirmed the absence of independent dietary predictors of dyslipidemia in this cohort. These null findings are biologically plausible within the context of ESRD, where lipid abnormalities are predominantly shaped by intrinsic metabolic disturbances rather than by diet alone.

In patients with advanced CKD and those on dialysis, uremic dyslipidemia arises mainly from impaired metabolism of triglyceride‐rich lipoproteins and dysfunctional HDL particles, driven by decreased lipoprotein lipase and hepatic lipase activity, elevated apolipoprotein C‐III, chronic inflammation, oxidative stress, and accumulation of uremic toxins, all of which alter lipoprotein clearance and composition independent of dietary inputs [7]. These pathophysiologic mechanisms are clearly documented in recent mechanistic overviews of lipid metabolism in CKD, which emphasize that altered TRL clearance and HDL dysfunction are central to dyslipidemia in this population, whereas the role of diet per se is limited compared with metabolic derangements inherent to renal failure [33].

Furthermore, although general population studies often demonstrate that dietary patterns, such as high intake of soluble fiber, omega‐3 PUFAs, and lower saturated fat intake, can favorably modify lipid profiles; these associations are weaker or inconsistent in CKD/ESRD patients. Meta‐analyses and dietary reviews suggest that although specific dietary components like long‐chain omega‐3 PUFAs may exert modest effects on triglycerides, standard renal dietary approaches have not consistently altered dyslipidemia outcomes, especially in the presence of metabolic dysfunction and inflammation characteristic of renal failure [33, 34, 35, 36]. For example, a recent narrative synthesis of dietary lipid recommendations in CKD highlighted that although PUFA intake shows some potential to improve TG levels, overall evidence is insufficient to substantively reduce dyslipidemia in advanced CKD without addressing underlying enzyme and metabolic abnormalities [37].

Additionally, protein‐energy wasting, loss of appetite, and the malnutrition‐inflammation complex syndrome have been proposed as important contributors to altered lipid metabolism in dialysis patients, potentially resulting in paradoxically low TC and LDL‐C levels and weakening the usual relationships between nutrient intake and lipid status observed in the general population. These phenomena are consistent with the broader concept of “reverse epidemiology,” whereby traditional cardiovascular risk factors may behave differently in patients receiving MHD. For example, lower cholesterol concentrations have been associated with poorer clinical outcomes, possibly reflecting underlying malnutrition and inflammation rather than a protective lipid profile [38]. However, because inflammatory biomarkers, such as C‐reactive protein (CRP), were not measured in the present study, the contribution of inflammation cannot be directly evaluated and should, therefore, be interpreted cautiously. Consequently, our findings should not be interpreted as evidence of a causal role of inflammation but rather as being consistent with mechanisms previously described in the literature. Future studies incorporating inflammatory biomarkers, alongside nutritional and dialysis‐related clinical parameters, are needed to better clarify the interplay between inflammation, nutritional status, and dyslipidemia in MHD patients. Although classic discussions of reverse epidemiology date back earlier, more recent clinical observations continue to suggest that metabolic disturbances associated with ESRD may exert a greater influence on lipid profiles than dietary factors alone [37].

### 4.6 Cardiovascular Risk Implications

CVD accounts for approximately 40%–50% of mortality among dialysis patients worldwide. Dyslipidemia contributes to endothelial dysfunction, accelerated atherosclerosis, and vascular calcification in CKD. However, the role of lipid‐lowering therapy after dialysis initiation remains complex. Large randomized controlled trials have demonstrated limited cardiovascular benefits from routine initiation of statins in prevalent dialysis patients, leading current KDIGO guidelines to recommend against universal statin initiation after dialysis onset while supporting continuation in patients who were already receiving lipid‐lowering therapy before starting dialysis [3]. Emerging evidence suggests that dialysis‐dependent patients with established atherosclerotic cardiovascular disease (ASCVD) may derive meaningful clinical benefit from statin treatment. A recent systematic review and meta‐analysis reported significant reductions in all‐cause mortality and major adverse cardiovascular events in this subgroup, supporting a targeted rather than universal approach to lipid‐lowering [39]. Therefore, cardiovascular risk management in dialysis patients should move beyond a blanket approach toward personalized decision‐making on the basis of individual cardiovascular history, comorbidities, age, inflammatory status, nutritional condition, and overall prognosis. In addition to individualized lipid‐lowering therapy, comprehensive lipid management should include optimization of ADAT, appropriate nutritional counseling consistent with CKD dietary recommendations, regular physical activity as tolerated, smoking cessation, and effective control of hypertension and diabetes. Periodic lipid monitoring and individualized cardiovascular risk assessment may further support timely therapeutic decision‐making. In resource‐limited settings, such as Bangladesh, where cardiovascular complications remain a major contributor to dialysis‐related mortality, integrated management strategies, including adequate dialysis delivery, nutritional optimization, and appropriate cardiovascular interventions, are essential to improve patient outcomes. Lipid‐lowering therapy should therefore be considered as part of a broader individualized cardiovascular risk reduction strategy rather than as a universal intervention for all dialysis patients.

## 5 Conclusion

This multicenter cross‐sectional study demonstrates a high prevalence of dyslipidemia among MHD patients in Bangladesh, affecting nearly four‐fifths of the population. The lipid profile is predominantly atherogenic, characterized by elevated triglycerides and reduced HDL‐C, indicating a markedly increased cardiovascular risk. The observed agreement between the TG/HDL‐C ratio and ATP III criteria further supports the use of the TG/HDL‐C ratio as a simple, cost‐effective screening tool, particularly in resource‐limited clinical settings. Importantly, the absence of significant associations between dietary macro‐ and micronutrient intake and dyslipidemia suggests that lipid abnormalities in this population are largely driven by underlying metabolic disturbances related to CKD and the hemodialysis process, rather than dietary factors alone. These findings underscore the need for a broader, more integrated clinical approach that includes optimizing ADAT, addressing inflammation, and implementing targeted cardiovascular risk reduction strategies. Overall, early identification and comprehensive management of dyslipidemia should be prioritized to mitigate the substantial cardiovascular burden in this high‐risk group. Future longitudinal studies incorporating inflammatory biomarkers and clinical outcomes are warranted to better understand causal mechanisms and guide evidence‐based, context‐specific interventions.

## 6 Strengths and Limitations

The multicenter design, comprehensive dietary assessment, and dual dyslipidemia classification methods strengthen the validity of this study. However, its cross‐sectional nature limits causal inference, and reliance on self‐reported dietary data may introduce recall bias. In addition, participants were recruited using a convenience sampling approach from selected dialysis centers, which may have introduced selection bias and limited the generalizability of the findings to the broader Bangladeshi ESRD population. Although recruitment from four geographically distinct districts enhanced sample diversity, future studies employing probability‐based sampling and larger nationally representative cohorts are warranted. The absence of inflammatory biomarkers, such as CRP, also limited the ability to directly evaluate the relationship between inflammation and dyslipidemia.

## 7 Future Directions

Future longitudinal studies should examine the prognostic value of TG/HDL‐C–based dyslipidemia in predicting cardiovascular events and mortality in Bangladeshi MHD patients. Integration of inflammatory markers, ADAT indices, and longitudinal nutritional assessments would further clarify the complex interplay between metabolism, nutrition, and cardiovascular risk in ESRD.

## Author Contributions

**Marium Sultana**: conceptualization, investigation, formal analysis, methodology, data curation, writing – original draft, validation. **Tanjina Rahman**: conceptualization, investigation, formal analysis, methodology, data curation, writing – original draft, validation. **Md. Abdul Halim**: conceptualization, investigation, formal analysis, methodology, data curation, writing – original draft, validation. **Md. Ariful Islam**: conceptualization and study design. All authors were involved in data curation, formal analysis, writing – original draft preparation, and writing – review and editing. All authors have read and confirmed the final draft of the manuscript.

## Funding

The authors have nothing to report.

## Conflicts of Interest

The authors declare no conflicts of interest.

## Data Availability

The datasets analyzed during the current study are available from the corresponding author upon reasonable request.
